# Temporal Changes of CB1 Cannabinoid Receptor in the Basal Ganglia as a Possible Structure-Specific Plasticity Process in 6-OHDA Lesioned Rats

**DOI:** 10.1371/journal.pone.0076874

**Published:** 2013-10-08

**Authors:** Gabriela P. Chaves-Kirsten, Caio H. Y. Mazucanti, Caroline C. Real, Bruna M. Souza, Luiz R. G. Britto, Andréa S. Torrão

**Affiliations:** 1 Laboratory of Neuronal Communication, Department of Physiology and Biophysics, University of São Paulo, São Paulo, Brazil; 2 Laboratory of Molecular Neuropharmacology, Department of Pharmacology, University of São Paulo, São Paulo, Brazil; 3 Laboratory of Cellular Neurobiology, Department of Physiology and Biophysics, University of São Paulo, São Paulo, Brazil; Karolinska Inst, Sweden

## Abstract

The endocannabinoid system has been implicated in several neurobiological processes, including neurodegeneration, neuroprotection and neuronal plasticity. The CB1 cannabinoid receptors are abundantly expressed in the basal ganglia, the circuitry that is mostly affected in Parkinson’s Disease (PD). Some studies show variation of CB1 expression in basal ganglia in different animal models of PD, however the results are quite controversial, due to the differences in the procedures employed to induce the parkinsonism and the periods analyzed after the lesion. The present study evaluated the CB1 expression in four basal ganglia structures, namely striatum, external globus pallidus (EGP), internal globus pallidus (IGP) and substantia nigra pars reticulata (SNpr) of rats 1, 5, 10, 20, and 60 days after unilateral intrastriatal 6-hydroxydopamine injections, that causes retrograde dopaminergic degeneration. We also investigated tyrosine hydroxylase (TH), parvalbumin, calbindin and glutamic acid decarboxylase (GAD) expression to verify the status of dopaminergic and GABAergic systems. We observed a structure-specific modulation of CB1 expression at different periods after lesions. In general, there were no changes in the striatum, decreased CB1 in IGP and SNpr and increased CB1 in EGP, but this increase was not sustained over time. No changes in GAD and parvalbumin expression were observed in basal ganglia, whereas TH levels were decreased and the calbindin increased in striatum in short periods after lesion. We believe that the structure-specific variation of CB1 in basal ganglia in the 6-hydroxydopamine PD model could be related to a compensatory process involving the GABAergic transmission, which is impaired due to the lack of dopamine. Our data, therefore, suggest that the changes of CB1 and calbindin expression may represent a plasticity process in this PD model.

## Introduction

The cannabinoid compounds are lipophilic molecules which exert their effects by binding to specific membrane receptors [1]. Two types of cannabinoid receptors, both coupled to G proteins, have been identified (CB1 and CB2). These two receptors control peripheral and central functions [2,3]. In addition, more recently it has been shown that cannabinoids can also bind to other types of receptors, such as the transient receptor potential vanilloid-1 (TRPV1) [4] and peroxisome proliferator-activated receptor (PPAR) family of nuclear receptors [5]. Endocannabinoids are usually released from target neurons and act as retrograde messengers that regulate synaptic transmission. The activation of presynaptic CB1 results in inhibition of the release of some neurotransmitters, such as glutamate and γ-aminobutyric acid (GABA) [6,7].

High amounts of CB1 are found in the basal ganglia [substantia nigra (SN), globus pallidus (GP), entopeduncular nucleus and lateral striatum], cerebellum, and hippocampus [1,3,8]. In the striatum, the highest expression of CB1 occurs in its dorsolateral portion [9] mainly in the terminals of cortical projection neurons and in GABAergic interneurons. In SN, CB1 receptors are located in the terminals of spiny striatal neurons in the pars reticulata (SNpr). Dopaminergic projections from SN innervate the striatal neurons that express CB1 and the dopamine receptors D1 and D2. The D2 and CB1 receptors share a group of G proteins, indicating a convergence of their transduction mechanisms. On the other hand, the activation of D1 receptors, mediated by adenylyl cyclase, can be completely blocked by CB1 stimulation. In the medial portion of globus pallidus (also known as internal globus pallidus – IGP) and in the SNpr it is well established that the activation of CB1 reduces the release of both GABA and glutamate from terminals originating in the striatum and subthalamic nucleus, respectively [10–14]. In addition to the high expression of CB1 in the basal ganglia, endocannabinoid ligands and their synthesizing and degrading enzymes are also particularly abundant in those structures [15].

Parkinson’s disease (PD) is a neurodegenerative disorder associated with loss of 50 to 70% of dopaminergic neurons in the SN pars compacta (SNpc), which has been extensively investigated in regard to its behavioral and molecular aspects, both in animal models and humans [16,17]. The loss of dopamine in the projection area of the SNpc neurons leads to a functional change in the complex circuitry of the basal ganglia [18], which results in an excessive inhibition of motor systems [19]. The high density of CB1 receptors and endocannabinoids found in the basal ganglia has been a much explored research field that seeks to understand the relation between PD and the cannabinoid system, which has led to prospects for cannabinoid therapies. Indeed, several studies have shown the participation of cannabinoid system in different kinds of neurodegenerative diseases and suggested its neuroprotective properties [20–23]. A recent study with humans, for instance, showed a marked decrease of CB1 in the SN of PD patients, concomitant with a slight increase in dopaminergic projection areas [24]. Delta9-tetrahydrocannabinol (delta9-THC) is able to reverse the decreased dopaminergic transmission in the basal ganglia of a mice model of PD [25].

Studies employing different animal models of PD, such as 6-hydroxydopamine (6-OHDA) injections in basal ganglia or intracerebroventricular in rats [26–29], intraperitoneal injections of heroin contaminant 1-methyl-4-phenyl-1,2,3,6-tetrahydropyridine (MPTP) in marmosets and mice [30], and specific park genes deleted- mice [31], for example, have also reported changes of CB1 expression in the basal ganglia. However, the data are heterogeneous and often conflicting. Those heterogeneous results seem to depend on the procedures employed to induce parkinsonism and the periods analyzed after the lesion [32].

Temporal variation of CB1 expression during PD progression is poorly understood. Furthermore, most studies are devoted to evaluate CB1 variation focusing on the striatum and SN, and giving minor attention to the other basal ganglia structures, such as external and internal globus pallidus (EGP and IGP, respectively – [33]). Therefore, the aim of this study was to analyze the time course of CB1 receptor expression by immunoshistochemistry in four structures of basal ganglia, namely striatum, EGP, IGP, and SN, in the PD model of unilateral intraestriatal injections of 6-OHDA in rats. The 6-OHDA injection directly into the striatum causes retrograde dopaminergic degeneration [16]. We also evaluated the expression of tyrosine hydroxylase (TH), glutamic acid decarboxylase (GAD), parvalbumin, and calbindin, by immunoshistochemistry and/or immunoblotting, in an attempt to establish relationships between the cannabinoid system and the dopaminergic and GABAergic systems.

## Materials and Methods

### Animals

Seventy-days-old male Wistar rats weighting 230-280g at the beginning of the experiments, provided by the central animal house of the Institute of Biomedical Sciences, University of São Paulo, were used in this study. The animals were housed in groups of 3–4 animals per cage, maintained at 23 °C ± 2, in a 12 h light/12 h dark cycle (lights on at 06:00 h), with free access to food and water. The experiments were carried out in accordance with the guidelines of the Brazilian College for Animal Experimentation (COBEA) and were approved by The Ethics Committee for Animal Research of the Institute of Biomedical Sciences, University of São Paulo (Protocol n° 27/68/2), which are in accordance with the procedures of national and international care of animals used in scientific research.

Independent groups of rats were used to investigate the time course effects of 6-OHDA striatal injection on protein levels by immunohistochemical and immunoblotting analysis.

### Surgical procedure and unilateral injection of 6-OHDA

Rats were anesthetized with 2,2,2-tribromoethanol (1 mL/100 g -250 mg/kg i.p. - Sigma®) and placed in a stereotaxic frame (David Kopf Instruments). After craniotomy, two 0.5 µl injections containing 6 µg of 6-OHDA in saline and 0.3% ascorbic acid (totalizing a final dose of 12 µg) were made in the right striatum by way of glass micropipettes coupled to a pressure system, in the following coordinates: (1) AP: bregma, ML: -2.7, and DV: -4.5mm, (2) AP: -0.5, ML: -3.4, and DV: 4 mm [33]. Once the infusion was finished, the micropipette was maintained still in the infused region for approximately one minute to avoid solution reflux, and then it was slowly removed from the brain. The incision was then sutured and treated with Fibrase® (Pfizer) in order to improve healing. The brains of PD-induced rats were then analyzed after 1, 5, 10, 20, and 60 days of the 6-OHDA injections (days post-lesion, DPL). The basal ganglia structures of the lesioned side (right) were always compared to their corresponding in the intact side (left).

### Transcardiac perfusion and Immunohistochemistry

PD-induced rats were deeply anesthetized with ketamine (5 mg/100 g of body weight, im) and xylazine (1 mg/100 g of body weight, im), and perfused through the left ventricle with phosphate-buffered saline and 4% paraformaldehyde in 0.1 M phosphate buffer (PB, pH 7.4). The brains were dissected out and after 4-6 hours post-fixation in paraformaldehyde solution they were transferred to a 30% sucrose PB solution to ensure cryoprotection. Coronal sections (30 µm) were then obtained on a sliding microtome and collected in PB.

Sections containing substantia nigra (SN), striatum and globus pallidus (GP) regions were incubated with rabbit polyclonal antiserum anti-CB1 (Cayman, Ann Arbor, MI, USA, 1:1,000), a mouse monoclonal anti-tyrosine hydroxylase antibody (TH; Chemicon, Temecula, CA, USA, 1:1,000) as an indicator of dopaminergic neurons in basal ganglia; a mouse monoclonal anti-parvalbumin antibody (Sigma®; 1: 1,000), and a mouse monoclonal anti-calbindin antibody (Sigma®; 1: 1,000), as indicators of GABAergic neurons in striatum, in PB containing 0.3% Triton X-100 plus 5% of normal goat or donkey sera. Parvalbumin is known to be present in GABAergic neurons in the basal ganglia [34,35]. Immunolabeling with peroxidase methods was conducted as previously described [36]. Controls for CB1 staining included: (1) omission of the primary antibody and (2) preadsorption of the primary antibody overnight with a 10-fold excess of the corresponding control peptide for CB1 (Cayman).

Digital images were acquired using a Nikon E1000 microscope (Melville, NY, USA) and Nikon DMX1200 digital camera (Nikon Imaging Software ACT-U). Images from striatum were captured between bregmas 1.80 and -0.36; from EGP between bregmas -0.24 and -3.12; from IGP between bregmas -1.92 and -2.76; and from SNpc and SNpr between bregmas -4.56 to -6.60 (4-8 sections/brain/structure; n= 4-5 per DPL) [33]. All structures were analyzed by obtaining images at certain intervals between each other in order to represent its entire length, thus ensuring an overview of each structure. Representative images were mounted with Adobe Photoshop (Adobe Systems Inc., Mountain View, CA, USA).

The TH expression was quantified in the striatum (6 sections) and SNpc (6-8 sections) as an indicator of the lesion extension. Parvalbumin and calbindin were quantified in the striatum (6 sections), parvalbumin in dorsolateral striatum and calbindin in ventromedial striatum. CB1 receptor expression was quantified in the striatum (6 sections), EGP (4 sections), IGP (4 sections), and SNpr (6 sections), all carried out with ImageJ (NIH/USA). For TH in SNpc, CB1 in EGP, IGP and SNpr, we measured the integrated density of immunolabeling and of the background (nonlabeled areas) from each slice. The background integrated density was deduced from the integrated density quantification of the labeled areas, to obtain a labeling index reflecting the signal-to-noise ratio. For CB1 immunostaining in the striatum the average of the integrated density of three areas of 2500 µm^2^ was conducted. For parvalbumin and calbindin in the striatum, positive cells were directly counted. After the quantification blind researchers to the experimental conditions (side and days after lesion) reviewed the data. The resulting indexes were always normalized to the controls.

### Immunoblotting

At the same time points described above, the rats (n=6-8 per group) were sacriﬁced by cervical dislocation, and the SN (bregma -4.56 to -6.60 below colliculus approximately), and striatum (bregma +2.28 to -1.32, approximately) [33] were rapidly collected. The immunoblotting assay was conducted as previously described [36]. In short, samples containing 30–40 µg protein were subjected to a 10% acrylamide gel containing sodium dodecyl sulfate and electrotransferred to nitrocellulose membranes using a Trans-Blot cell system (Bio-Rad). The membranes were then blocked for at least 2 hr and incubated overnight with the antibodies against TH or a rabbit policlonal antibody anti-GAD (Millipore, Temecula, CA, USA). Loading control with β-actin was conducted in all experiments by using a mouse monoclonal antibody against β-actin (Sigma, St. Louis, MO). Optical density obtained from TH and GAD was ﬁrst normalized in relation to the corresponding β-actin bands in each experiment. Subsequently, the normalized data were treated to evaluate protein levels changes in the experimental side in relation to their controls for each time point.

As described for immunohistochemistry, the TH protein levels were quantified by the immunoblotting method as an indication of the lesion extension. GAD levels were investigated in the striatum and SN to obtain indirect data on GABAergic neurons. After the quantification blind researchers to the experimental conditions (side and days after lesion) reviewed the data.

### Statistical Analysis

Quantitative data were submitted to statistical analysis using GraphPad Prism 5 (San Diego, CA). The comparisons between control and experimental sides from each group were made using the Paired Student’s t-test. The comparisons between all groups were made using the One-Way ANOVA test, followed by Tukey’s post-test when appropriate. For all tests described, the significance value adopted was p≤0.05. All data were normalized to the controls and are expressed as means ± SEM.

## Results

### Time course expression of TH, GAD, parvalbumin, calbindin and CB1 in the basal ganglia of 6-OHDA injected rats

**Figure 1:**
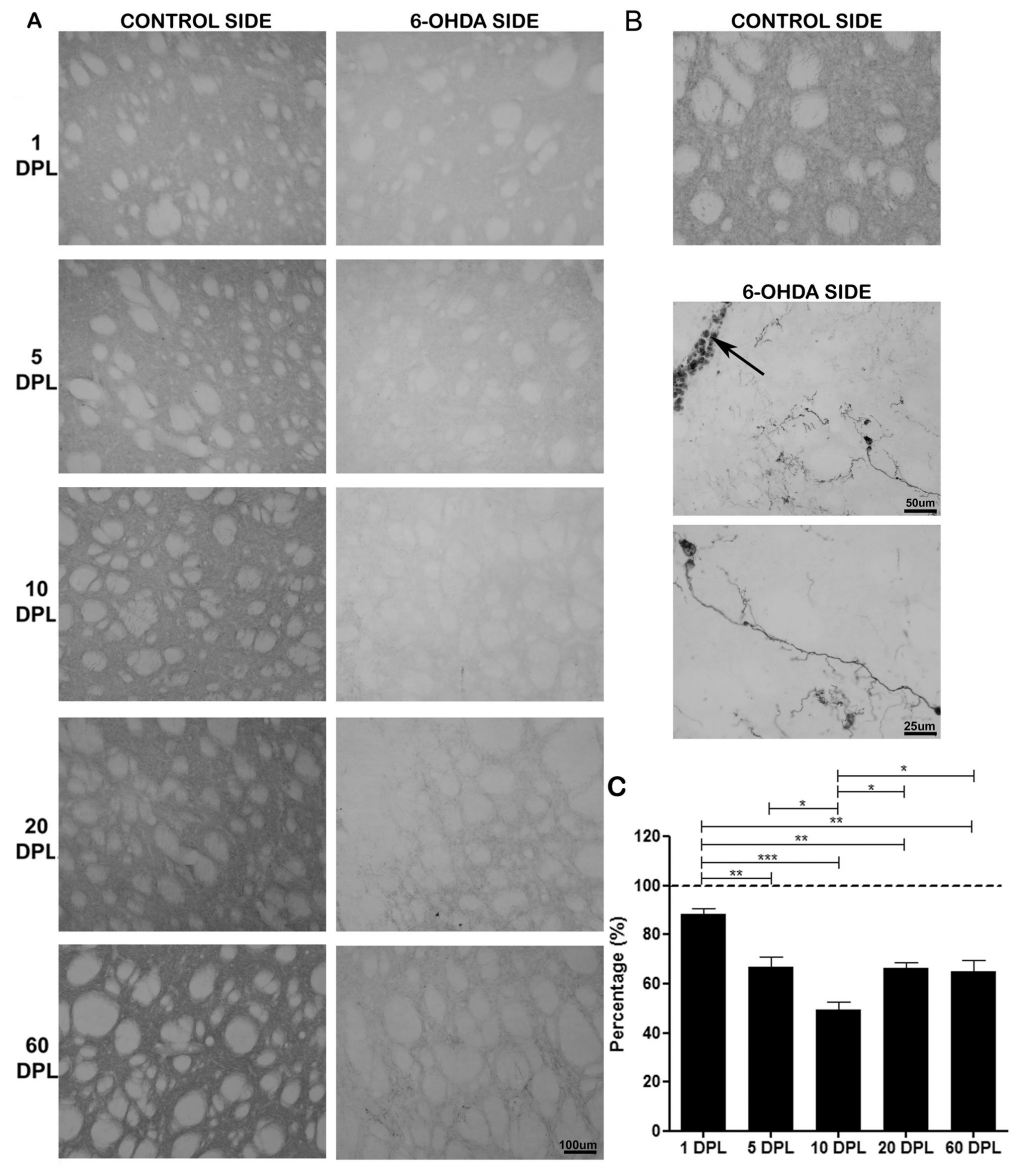
Temporal changes of TH levels in the striatum of PD-induced rats. TH levels in striatum of rats submitted to unilateral intrastriatal injections of 6-OHDA after 1, 5, 10, 20, and 60 days post-lesion (DPL). (A) Digital images of coronal sections of the dorsolateral striatum shows a decrease in the expression of TH on the 6-OHDA side, with a peak in 10 DPL (B) Digital images of coronal sections of the dorsolateral striatum at 60 DPL; the arrow indicates the site of infusion. Note the emergence of TH+ cells in the experimental side. (C) Semi-quantitative analysis of TH staining represented as percentage. Control side (dashed line). Statistical analysis by ANOVA (one way) with Tukey post-test. * p <0.05, ** p <0.01 and *** p <0.001.

**Figure 2:**
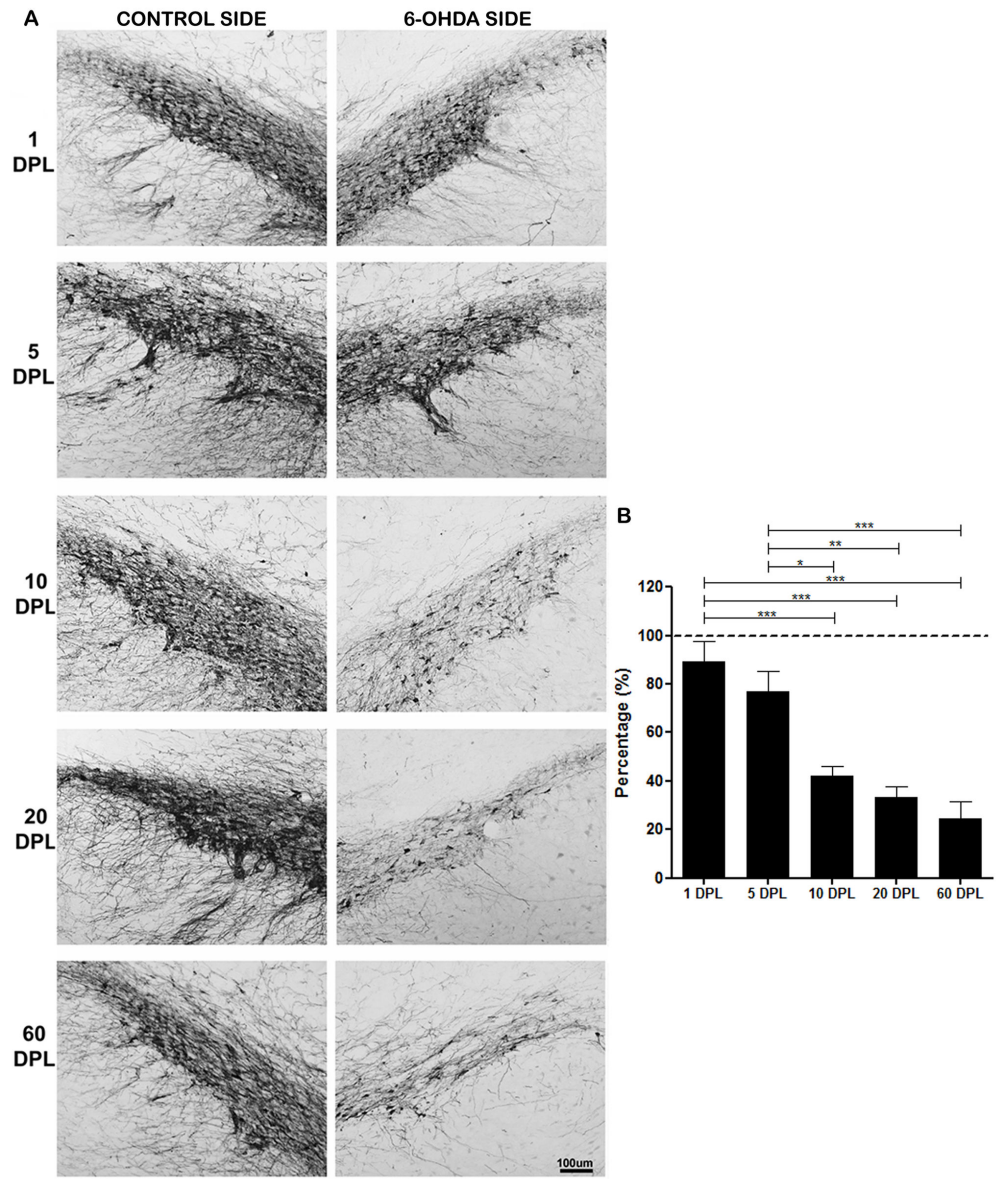
Temporal changes of TH expression in the SNPc of PD-induced rats. TH expression in SNpc of rats submitted to unilateral intrastriatal injections of 6-OHDA after 1, 5, 10, 20 and 60 days post-lesion (DPL). (A) Digital images of coronal sections of the SNpc show a progressive decreased TH expression in 6-OHDA side. (B) Semi-quantitative analysis of TH staining represented as percentage. Control group (dashed line). Statistical analysis by ANOVA (one way) with Tukey post-test. * p <0.05, ** p <0.01 and *** p <0.001.

**Figure 3:**
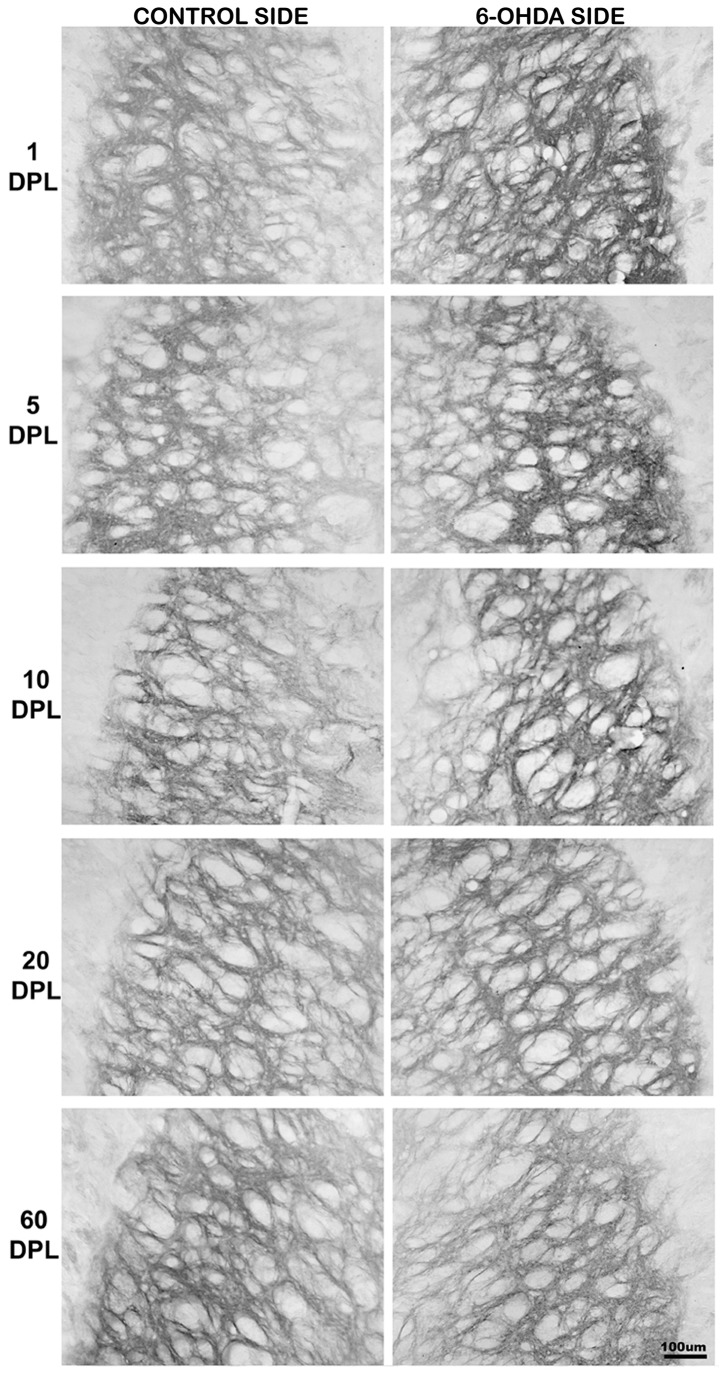
Temporal changes of CB1 expression in the EGP of PD-induced rats. CB1 expression in external globus pallidus (EGP) of rats submitted to unilateral intrastriatal injections of 6-OHDA after 1, 5, 10, 20, and 60 days post-lesion (DPL). Digital images of coronal sections of the EGP show an increase of CB1 expression in the experimental side at earlier time points.

**Figure 4:**
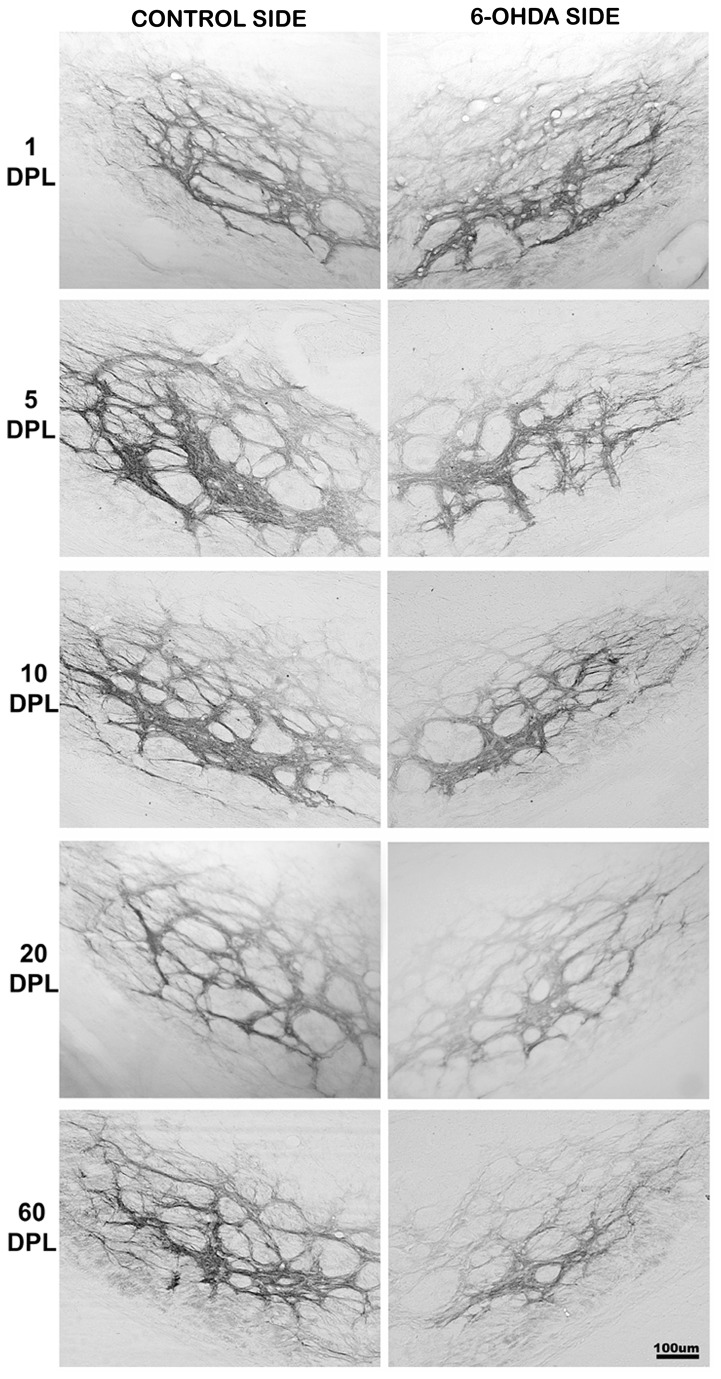
Temporal changes of CB1 expression in the IGP of PD-induced rats. CB1 expression in internal globus pallidus (IGP) of rats submitted to unilateral intrastriatal injections of 6-OHDA after 1, 5, 10, 20 and 60 days post-lesion (DPL). Digital images of coronal sections of the IGP show an increase in the CB1 expression in the experimental side at 1 DPL. From 5 to 60 DPL, there was a gradual decrease in the CB1 expression.

**Figure 5:**
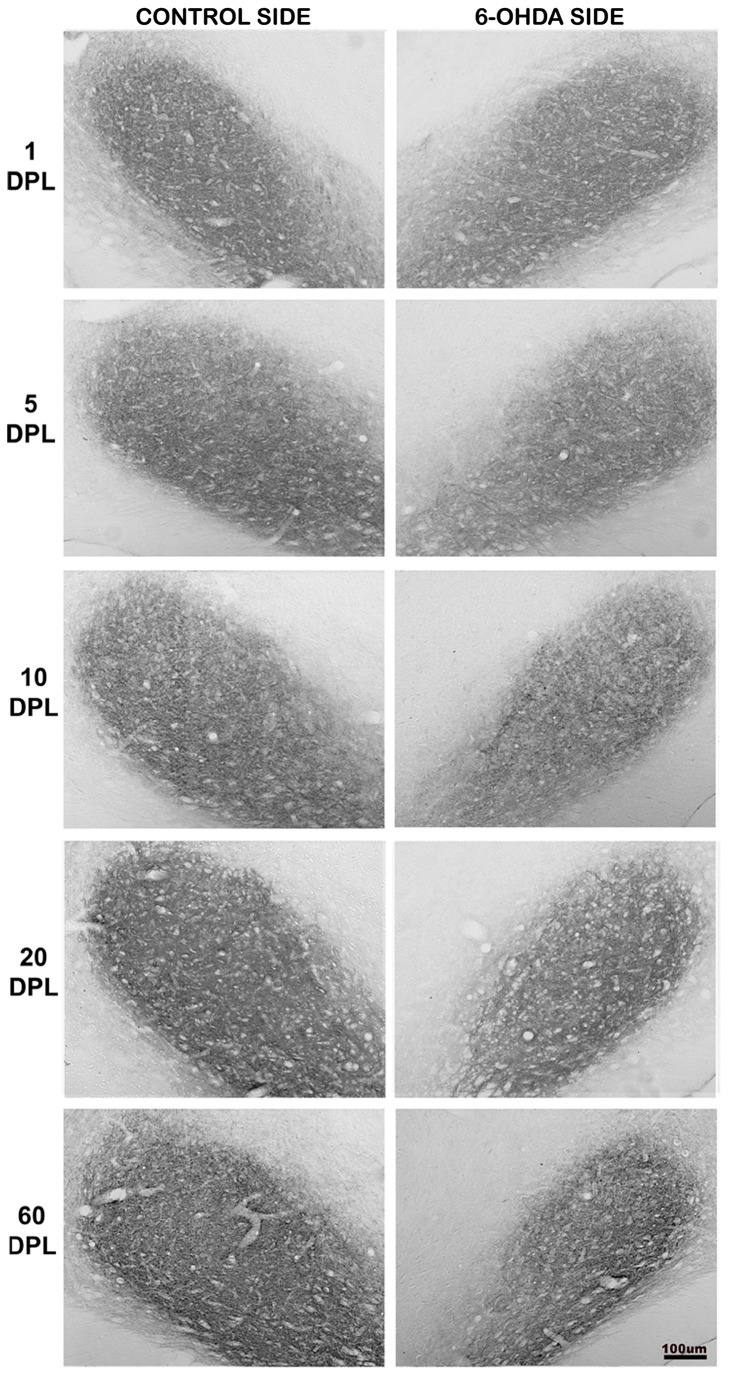
Temporal changes of CB1 expression in the SNpr of PD-induced rats. CB1 expression in the substantia nigra pars reticulata (SNpr) of rats submitted to unilateral intrastriatal injections of 6-OHDA after 1, 5, 10, 20 and 60 days post-lesion (DPL). Digital images of coronal sections of the SNpr show a decrease in the CB1 expression in the experimental side from 5 DPL to 60 DPL.

The analysis of TH, GAD, parvalbumin, calbindin and CB1 protein expression is summarized in Table 1 (immunoblotting) and in Table 2 (immunohistochemistry), and described in detail below. Representative digital images are shown in Figures 1-5.

**Table 1:** Immunoblotting analysis of TH and GAD expression in the basal ganglia of rats after 1, 5, 10, 20, and 60 days of the 6-OHDA injections (days post-lesion, DPL).

Structure	Protein	1 DPL CONT Mean (±SEM)	1 DPL EXP Mean (±SEM)	5 DPL CONT Mean (±SEM)	5 DPL EXP Mean (±SEM)	10 DPL CONT Mean (±SEM)	10 DPL EXP Mean (±SEM)	20 DPL CONT Mean (±SEM)	20 DPL EXP Mean (±SEM)	60 DPL CONT Mean (±SEM)	60 DPL EXP Mean (±SEM)
Striatum	TH	1.00 (±0.08)	0.90 (±0.10)	1.00 (±0.09)	0.72* (±0.08)	1.00 (±0.07)	0.34**** (±0.06)	1.00 (±0.07)	0.41**** (±0.06)	1.00 (±0.04)	0.50**** (±0.05)
Striatum	GAD	1.00 (±0.18)	1.09 (±0.20)	1.06 (±0.12)	1.02 (±0.17)	1.03 (±0.09)	0.98 (±0.10)	1.06 (±0.09)	1.25 (±0.10)	1.02 (±0.07)	1.00 (±0.14)
SN	TH	1.00 (±0.08)	0.99 (±0.06)	1.00 (±0.08)	0.98 (±0.07)	1.00 (±0.07)	0.71** (±0.07)	1.00 (±0.12)	0.54** (±0.11)	1.00 (±0.07)	0.55*** (±0.03)
SN	GAD	1.00 (±0.05)	0.86 (±0.09)	1.00 (±0.14)	1.04 (±0.15)	1.00 (±0.07)	0.96 (±0.08)	1.00 (±0.16)	1.03 (±0.18)	1.03 (±0.13)	1.15 (±0.14)

(DPL) days post-lesion, (CONT) Control side, (EXP) experimental side, (SN) substantia nigra. Statistical analysis by Paired Student’s t test, (*p<0.05; **p<0.01; ***p<0.001, ****p<0.0001 vs control side for each group)

**Table 2:** Immunohistochemistry analysis of TH, CB1, parvalbumin and calbindin expression in the basal ganglia of rats after 1, 5, 10, 20, and 60 days of the 6-OHDA injections (days post-lesion, DPL).

Structure	Protein		1 DPL CONT Mean (±SEM)	1 DPL EXP Mean (±SEM)	5 DPL CONT Mean (±SEM)	5 DPL EXP Mean (±SEM)	10 DPL CONT Mean (±SEM)	10 DPL EXP Mean (±SEM)	20 DPL CONT Mean (±SEM)	20 DPL EXP Mean (±SEM)	60 DPL CONT Mean (±SEM)	60 DPL EXP Mean (±SEM)
DL Striatum	CB1		1.00 (±0.05)	1.03 (±0.05)	1.00 (±0.06)	1.02 (±0.07)	1.00 (±0.02)	1.03 (±0.02)	1.00 (±0.03)	0.99 (±0.03)	1.00 (±0.03)	0.99 (±0.04)
EGP	CB1		1.00 (±0.06)	1.45** (±0.12)	1.00 (±0.02)	1.29** (±0.07)	1.00 (±0.05)	1.13 (±0.05)	1.00 (±0.03)	1.00 (±0.04)	1.00 (±0.05)	0.86 (±0.05)
IGP	CB1		1.00 (±0.08)	1.52** (±0.10)	1.00 (±0.06)	0.83* (±0.09)	1.00 (±0.02)	0.74** (±0.07)	1.00 (±0.05)	0.41**** (±0.03)	1.00 (±0.04)	0.42*** (±0.08)
SNpr	CB1		1.00 (±0.03)	0.92 (±0.04)	1.00 (±0.09)	0.84** (±0.08)	1.00 (±0.06)	0.65*** (±0.05)	1.00 (±0.04)	0.60*** (±0.05)	1.00 (±0.02)	0.61** (±0.09)
DL Striatum	PV		1.00 (±0.07)	1.03 (±0.03)	1.00 (±0.09)	0.94 (±0.04)	1.00 (±0.09)	0.90 (±0.11)	1.00 (±0.04)	0.99 (±0.06)	1.00 (±0.02)	1.09 (±0.08)
VM Striatum		Calb	1.00 (±0.03)	1.21** (±0.05)	1.00 (±0.02)	1.21** (±0.02)	1.00 (±0.03)	1.01 (±0.03)	1.00 (±0.03)	1.00 (±0.03)	1.00 (±0.01)	0.99 (±0.03)
DL Striatum		TH	1.00 (±0.04)	0.90* (±0.02)	1.00 (±0.02)	0.67*** (±0.05)	1.00 (±0.04)	0.48*** (±0.02)	1.00 (±0.02)	0.66*** (±0.02)	1.00 (±0.04)	0.63*** (±0.05)
SNpc	TH		1.00 (±0.05)	0.89 (±0.08)	1.00 (±0.05)	0.77* (±0.08)	1.00 (±0.07)	0.42** (±0.04)	1.00 (±0.04)	0.33*** (±0.05)	1.00 (±0.07)	0.25** (±0.07)

(DPL) days post-lesion, (CONT) Control side, (EXP) experimental side, (DL) dorsolateral, (VM) ventromedial, (EGP) external globus pallidus, (IGP) internal globus pallidus, (SNpr) substantia nigra pars reticulata, (SNpc) substantia nigra pars compacta. Statistical analysis by Paired Student’s t test, (*p<0.05; **p<0.01; ***p<0.001; ****p<0.0001 vs control side for each group)

### TH expression in the basal ganglia decreases over time in 6-OHDA injected rats

In general, 6-OHDA produced a decreased TH immunoreactivity in the injured striatum and SNpc compared to control side. In the striatum, the 6-OHDA produced a gradual decrease in the TH immunoreactivity, which was more pronounced at 10 DPL, followed by a slight increase at 20 and 60 DPL (Table 2, Figure 1A). The mean percentage of TH expression changes in the immunohistochemistry, as well as the p values of ANOVA between DPLs over time in the striatum are shown in Figure 1C. In these later periods, mainly at 60 DPL, we observed TH-positive perikarya in the injured striatum, when compared to the control side, which normally exhibits TH immunoreactivity exclusively in the neuropil. These cells were strongly stained for TH and its frequency was higher in sites close to the infusion of 6-OHDA (Figure 1B). Semi-quantitative analysis of the immunoblotting (Table 1) corroborates those found in immunohistochemistry.

In the SNpc a gradual and progressive decrease of TH immunoreactivity was observed in immunohistochemistry (Table 2, Figure 2A) and in immunoblotting (Table 1). The mean percentage of TH expression changes in the immunohistochemistry, as well as the p values of ANOVA between DPLs over time in the SNpc are shown in Figure 2B.

### Time course expression of CB1 changes differentially in basal ganglia structures of 6-OHDA injected rats

The CB1 analyses were performed only for immunohistochemistry, because a complex and distinct structure-related variation of its expression was observed and the immunoblotting analysis would not allow a detailed spatial resolution.

Our studies showed that CB1 receptor was present exclusively in neuropil in all structures of the basal ganglia analyzed. In the striatum, we found higher densities of CB1 in its dorsolateral portion. In the GP, CB1 was expressed throughout the structure, and in the SN, was restricted to the SNpr. The immunolabelling for CB1 was very specific, as we had no immunostaining in our negative controls, namely the omission of primary antibody and preadsorption of the primary antibody with the corresponding control peptide for CB1 (data not shown).

**Figure 6:**
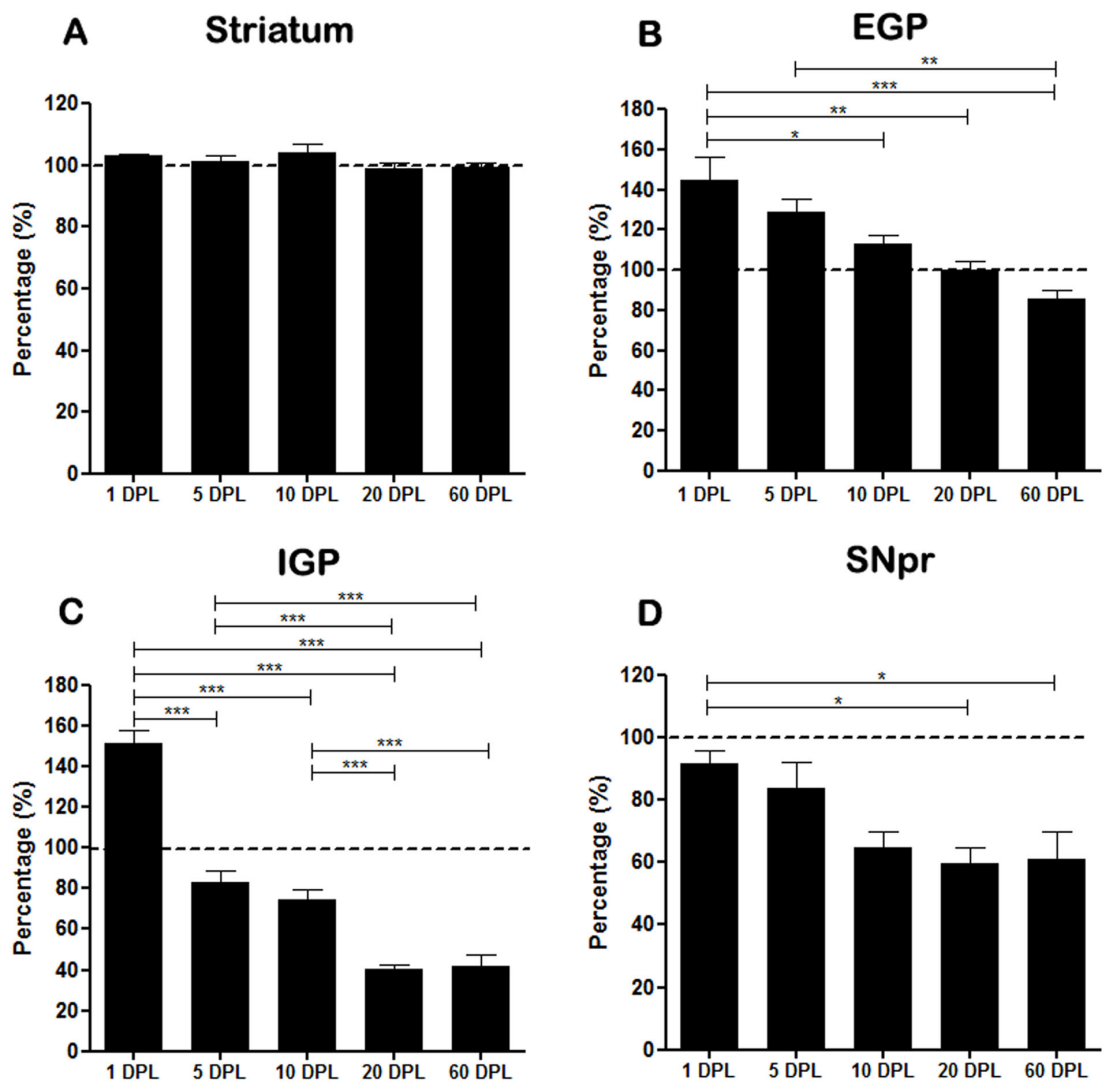
Semi-quantitative analysis of CB1 staining in basal ganglia of PD-induced rats. The quantification is represented by percentage in (A) striatum, (B) external globus pallidus (EGP), (C) internal globus pallidus (IGP) and (D) substantia nigra pars reticulata (SNpr) of rats submitted to unilateral intrastriatal injections of 6-OHDA after 1, 5, 10, 20, and 60 days post-lesion (DPL). Comparison between the percentage of experimental sides calculated from the control side to each group (dashed line). Statistical analysis by ANOVA (one way) with Tukey post-test. *p <0.05, **p<0.01 and *** p <0.001.

In the striatum, our data showed that there is no difference in the expression of CB1 in injured striatum in all DPL studied (Table 2 and Figure 6). However, in EGP the time course of CB1 expression showed a biphasic pattern, with an increase at short time points, which was gradually decreased at longer time ones (Table 2 and Figures 3 and 6). As for the EGP, the time course of CB1 expression in the IGP was biphasic, but with a different pattern. The increased expression of CB1 occurred only after 1 DPL, followed by a gradual decrease from 5 DPL (Table 2 and Figures 4 and 6). The SNpr exhibited a gradual decrease in CB1 immunoreactivity that seems to occur since the shorter time point (Table 2 and Figures 5 and 6).

The CB1 expression data after the lesions are summarized in Table 3.

**Table 3:** Summary of CB1 expression changes in the basal ganglia.

**Structure**	**1 DPL**	**5 DPL**	**10 DPL**	**20 DPL**	**60 DPL**
**Striatum DL**	=	=	=	=	=
**EGP**	↑↑↑↑	↑↑	↑	=	↓
**IGP**	↑↑↑↑	↓	↓↓	↓↓↓	↓↓↓
**SNpr**	=	↓	↓↓	↓↓↓	↓↓↓

(DPL) days post-lesion, (DL) dorsolateral, (EGP) external globus pallidus, (IGP) internal globus pallidus, (SNpr) substantia nigra pars reticulata; (

↑↑ ↑ ↑ /↓ ↓ ↓ ↓ very large increase/decrease; (↑↑ ↑ /↓ ↓ ↓ large increase/decrease; (↑↑ /↓ ↓ moderate increase/decrease; (↑ /↓ little increase/decrease; (=) no change. All in relation to control side for each group.

The data above were generated by comparisons between experimental and control sides for each DPL. We have also performed another statistical comparison (ANOVA) between the experimental side/control side ratios for each DPL, to evaluate the differences over time. The mean percentage of CB1 expression changes in the immunohistochemistry, as well as the p values of ANOVA between DPLs over time in the striatum, EGP, IGP, and SNpr are shown in Figure 6.

### 6-OHDA injection does not change the expression of GAD and parvalbumin, but increase the calbindin in the basal ganglia

As CB1 receptors are located in the axon terminals of GABAergic neurons in the basal ganglia [8], and Walsh et al. [28] described that the decrease of CB1 expression in SNpr could be an indirect toxic effect of 6-OHDA to GABAergic neurons, we have investigated the levels of GAD in the striatum and SN, and the immunolabeling for parvalbumin and calbindin in striatum (both immunolabeling known to be co-localized with GABAergic neurons in basal ganglia [34,35]), to verify a possible degeneration of these GABAergic neurons in the striatum caused by 6-OHDA. Semi-quantitative analysis of immunoblotting data showed no changes in GAD levels in the striatum and SN for all DPL studied (Table 1 and Figure S1). Our analysis showed no differences in the number of parvalbumin positive cells between the control and experimental striata (Table 2 and Figure S1).

Calbindin was observed mainly in cell body in the ventromedial portion. There was as an increase about 21% in the calbindin-positive cells for both 1 and 5 DPL (p<0.01 and p<0.001, respectively) (Table 2 and Figure S2).

## Discussion

Our results revealed a complex and distinct structure-specific variation of CB1 in the basal ganglia over time in 6-OHDA PD model. This variation was accompanied by an initial general TH reduction which is reversed by an increase at later periods in the striatum. No changes in the number of GABAergic neurons were observed in the striatum.

Several studies have shown the participation of the cannabinoid system in different neurodegenerative diseases and suggested its neuroprotective properties [21–23]. In this context, the high densities of CB1 receptors [1,8] and the endocannabinoid ligands found in the basal ganglia [15] have been explored in regard to PD, which has led to prospects for cannabinoid therapies.

First, to verify a possible relationship between the CB1 expression and the progression of PD, we analyzed the TH expression as a dopaminergic lesion marker. In general, our data support the literature data regarding the dopaminergic neurons death, since we observed a marked decrease of TH expression in the injured side, both in the striatum and SN. In the SN this decrease was gradual over the time points studied, and was more pronounced in the later ones, as described by several other studies (reviewed by [16,37,38]; [39,40]. On the other hand, in the striatum we observed a discrete increase in the TH levels after longer post-lesion periods (20 and 60 DPL), probably due to the increase of the striatal TH-staining intrinsic circuitry. Other studies have described a small intrinsic striatal dopaminergic circuitry [41], that shows a signiﬁcant increase in the number of TH positive neurons in the dopamine-depleted striatum [42]

There are few studies establishing a direct relationship between variation in the CB1 expression and the development of PD, as well as in animal models of PD. In addition, these studies, despite generating highly controversial data, are dedicated in most cases only to the striatum and/or SN [28,29,43].

First, it is important to note that the immunolabeling for CB1 in the basal ganglia found in our study showed a similar pattern of localization to other studies conducted with another CB1 antibody in immunohistochemistry [12] or autoradiography [8] methods. Our data show a decrease in CB1 receptor expression in the SN, starting at short periods after the 6-OHDA injection and gradually decreasing over time. In the striatum, however, no changes in the CB1 expression were observed, despite the reduction of TH. Interestingly, in the GP we observed a biphasic time course CB1 variation, with an initial increase of expression followed by a reduction. Moreover, this variation was distinct between the external and internal portions of the GP. In the EGP the increase in the CB1 expression remains until 10 days of the lesion and in the IGP it was only observed after one day post-lesion. In the case of earlier time points, we believe that the increased CB1 expression can be related to inflammatory events that occurs in basal ganglia due to infusion of 6-OHDA. In a mice model of neuroinflammation with peripheral administration of lipopolysaccharide (LPS), an increased CB1 mRNA in the hippocampus and brainstem was seen after 24h [44]. In the PD context, it was observed that the CB1 protects nigrostriatal dopaminergic neurons against MPTP neurotoxicity in mouse, by preventing microglia-derived oxidative damage, suggesting a close relationship between the cannabinoid system and neuroinflammatory events [45]

Decreased levels of CB1 mRNA in the striatum were found in the PD model induced by reserpine in rats [43] and Walsh et al. [28] described the reduction of CB1 expression in the SN over time (1-28 days after lesion) in the 6-OHDA model. Since the main CB1 location in SNpr is in the GABAergic terminals, the authors attributed the decrease of CB1 expression to an indirect toxic effect of 6-OHDA to GABAergic neurons, which undergo degeneration and consequently decrease the CB1expression. However, we did not find any differences in GAD levels in the striatum and SN, neither decrease in the number of parvalbumin and calbindin positive cells in the striatum, at any time points studied, which led us to suppose that the CB1 decrease appears to be unrelated to a possible degeneration of GABAergic neurons. Indeed, in the case of calbindin we observed an increased expression in the striatum at shorter post-lesion periods, which could be related to its function as Ca^2+^ buffering. Studies show that in damaging processes there is an increase of Ca^2+^ levels, and the calbindin may increase the activity to buffering its overload, thus protecting neurons from excitotoxic damage [46,47].

The decreased levels of CB1 mRNA were also found in post-mortem brains of PD patients [48]. A recent study employing a PET technique performed with specific CB1 radioligands reported a marked CB1 decrease in the SN of PD patients, concomitant with a slight increase of this receptor in dopaminergic projection areas [24].

A biphasic pattern of change in CB1 expression in the basal ganglia was observed in a previous study using mice with deletion of specific park genes [31,49]. In the early stages of asymptomatic PD (12 months of age) a desensitization/downregulation of CB1 occurs in GP and SN, particularly in the alpha-synuclein-deficient mice, which was associated to a dopaminergic dysfunction. In contrast, in later stages of PD (older than 12 months of age), characterized by a deep nigral degeneration associated to the appearance of parkinsonian symptoms, was related to a CB1 upregulation [49]. The expression of CB1 in the GP seems to be influenced by degenerative processes that affect the basal ganglia, like Huntington’s disease [50–52], and multiple sclerosis [53]. Although explored in other neurodegenerative diseases, the CB1 expression in the GP is still not well studied in animal models of PD. Here we show for the first time the variation in the CB1 levels in all four basal ganglia structures that express CB1 in the PD 6-OHDA model, including both GP segments. This information is essential to understand the involvement of CB1 in the basal ganglia circuitry in the PD condition.

The literature points to the involvement of cannabinoid system in the basal ganglia circuitry and that this system is changed in neurodegenerative diseases [11,49]. It seems that the endocannabinoid and dopaminergic systems exert a mutual control on each other. In PD, for example, it has been suggested that changes in the cannabinoid system may participate in symptom generation or as part of a compensatory mechanism to counteract the unbalance in the physiology of the basal ganglia due to the lack of dopamine [11]. Apart from the above-mentioned studies on the variations of CB1 expression, other studies showed changes in levels of endocannabinoids, such as anandamide [54] or changes in endocannabinoid membrane transporters and fatty acid amide hydrolase activity [55].

In relation to present data, the basal ganglia structure-specific time course variation in the CB1 expression could be a plastic response as part of a compensatory mechanism, as seen in studies of cannabinoid system in other pathways, such as retinal ablation [36], bilateral vestibular deafferentation [56], and in pilocarpine-induced epilepsy [57].

**Figure 7:**
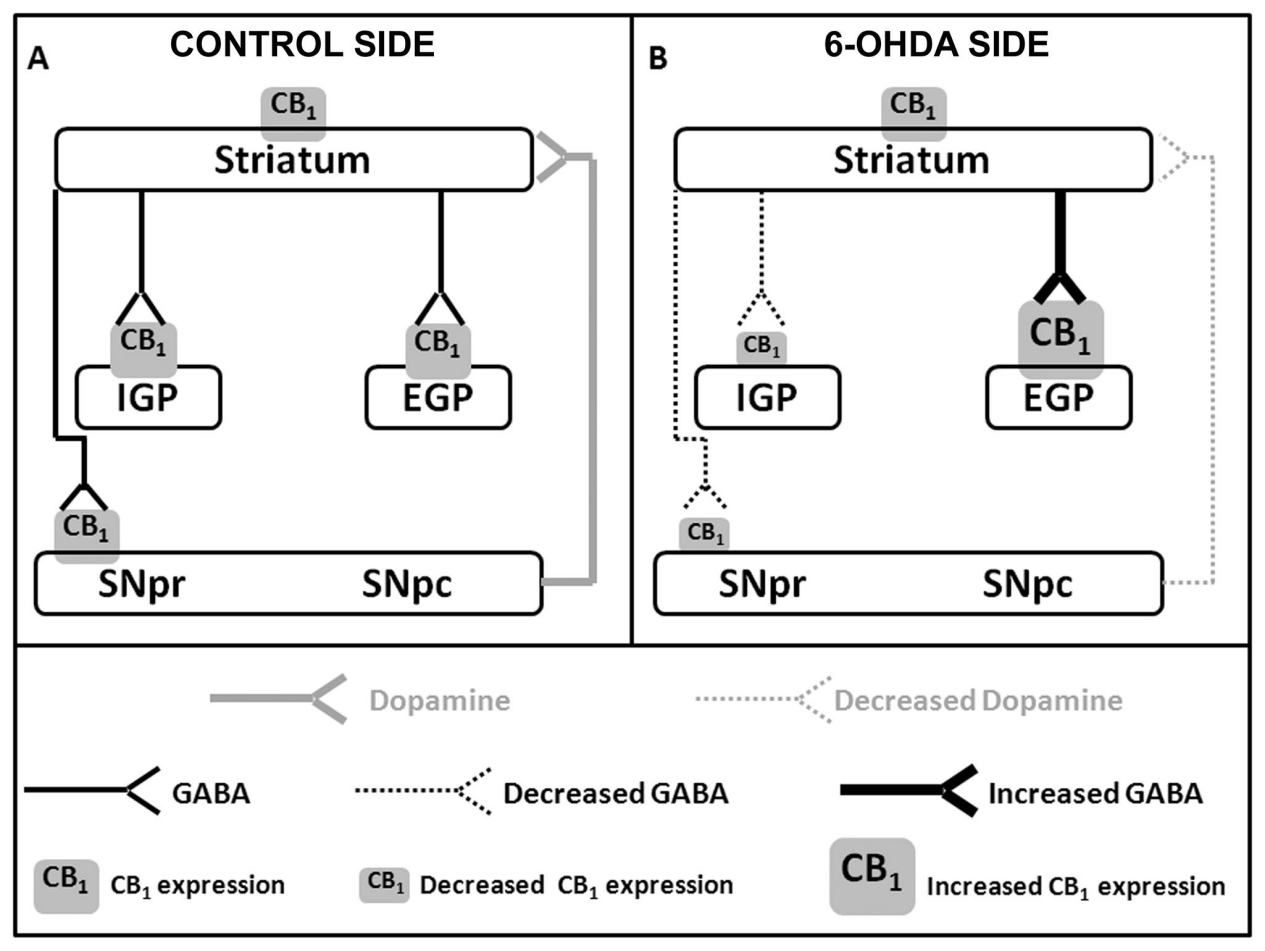
Scheme of CB1 expression in basal ganglia of PD-induced rats. Schematic representation of the CB1 receptors expression in the striatum, external globus pallidus (EGP), internal globus pallidus (IGP) and substantia nigra pars reticulata (SNpr). This scheme represents control side (A) and intrastriatal 6-OHDA injected side (B). Figure in A depicts the dopaminergic projection (full gray arrows) of the substantia nigra pars compact (SNpc) to the striatum, and GABAergic projections (full black arrows) of the striatum to EGP, IGP and SNpr, where CB1 receptors are found (gray squares). Figure in B represents the decreased dopaminergic (dotted gray arrows) and GABAergic (dotted black arrows) projections, the increased GABAergic projections (thicker full black arrows), increased CB1expression (bigger gray square), and the decreased CB1 expression (smaller gray square) after 6-OHDA injection. Our hypothesis is that the decrease of CB1 expression in the direct pathway (SNpr and IGP) could increase the GABAergic transmission, and the increase of CB1 expression in indirect pathway (EGP) could decrease the GABAergic transmission, which could together contribute to a restoration of normal thalamic activation. Both events may be a plastic response of the cannabinoid system due to the lack of dopamine.

The data obtained here on CB1 changes may be related to GABA-dependent transmission into the basal ganglia. The decreased GABAergic transmission to SNpr and IGP promotes an increase in neuronal activity in both structures, whereas the increase in GABAergic transmission generates a larger inhibition in EGP [19]. Under physiological conditions, the activation of pre-synaptic CB1 in GABAergic neurons inhibits the GABA release [6]. Our hypothesis is that the decrease of CB1 in SNpr and IGP in the 6-OHDA PD model could be a plastic response as an attempt to increase the GABAergic transmission which was altered by the dopaminergic degeneration. Following the same reasoning, increased CB1 expression in the EGP may lead to a lower release of GABA, which could recover part of the neuronal activity (Figure 7).

Even for the EGP it is important to remember that despite the initial CB1 increase in shorter DPL, at 60 DPL there is a tendency to a decreased expression of CB1. Transient variations in the CB1 receptor expression has been observed by our group in different situations in the chick optic tectum, such as during embryonic development [58] and after retinal ablation [36]. CB1 receptor variation seems to be common also in pilocarpine model of epilepsy, which exhibited a downregulation of hippocampal CB1 receptors in the acute phase, followed by compensatory upregulation and sprouting in the chronic phase of lobe temporal epilepsy [59]. Similar pattern of hippocampal CB1 receptor change was seen in pilocarpine-induced status epilepticus [57]. These data taken together suggest the involvement of changes in the CB1 expression in the neurodegenerative processes.

Finally, some authors describe that CB1 may increase local GABA levels in the EGP by reducing GABA reuptake from striatal afferents to that nucleus [13,60]. It is then possible that a decrease of CB1 in EGP at longer DPL is an attempt to decrease the release of GABA for this structure.

## Conclusion

In conclusion, our results indicate a complex modulation of CB1 expression in the basal ganglia over time after intrastriatal injections of 6-OHDA, an animal toxin-induced PD model largely investigated. This modulation is in agreement with data from transgenic PD animal models and even more important, data from PD patients. The fact that changes in CB1 expression are structure-specific, led us to speculate that our data may help to explain the participation of the cannabinoid system in a possible compensatory mechanism in PD, possibly related to neuronal plasticity. Furthermore, our data appear to validate the intrastriatal injection of 6-OHDA as a good PD model to study the cannabinoid system in PD.

## Supporting Information

Figure S1 **Temporal analysis of GAD levels in basal ganglia of PD-induced rats.** Semi-quantitative analysis from immunoblotting. Mean ratio of GAD densitometry density data in relation to beta-actin, comparing the experimental with control side from each DPL. Statistic analysis by Paired Student t test.
(TIF)

Figure S2 **Semi-quantitative analysis of calbindin and parvalbumin staining in basal ganglia of PD-induced rats.** The quantification is represented by percentage in the striatum of rats submitted to unilateral intrastriatal injections of 6-OHDA after 1, 5, 10, 20 and 60 days post-lesion (DPL). (A) Parvalbumin (PV) and (B) Calbidin. Comparison between the percentage of experimental sides calculated from the control side to each group (dashed line). Statistical analysis by ANOVA (one way) with Tukey post-test. *p <0.05, **p<0.01 and *** p <0.001.
(TIF)
