# Supplementary figures and images for: Temporal Changes of CB1 Cannabinoid Receptor in the Basal Ganglia as a Possible Structure-Specific Plasticity Process in 6-OHDA Lesioned Rats
Source: PLoS One. 2013 Oct 8;8(10):e76874. doi: 10.1371/journal.pone.0076874 (PMC3792868; doi:10.1371/journal.pone.0076874)

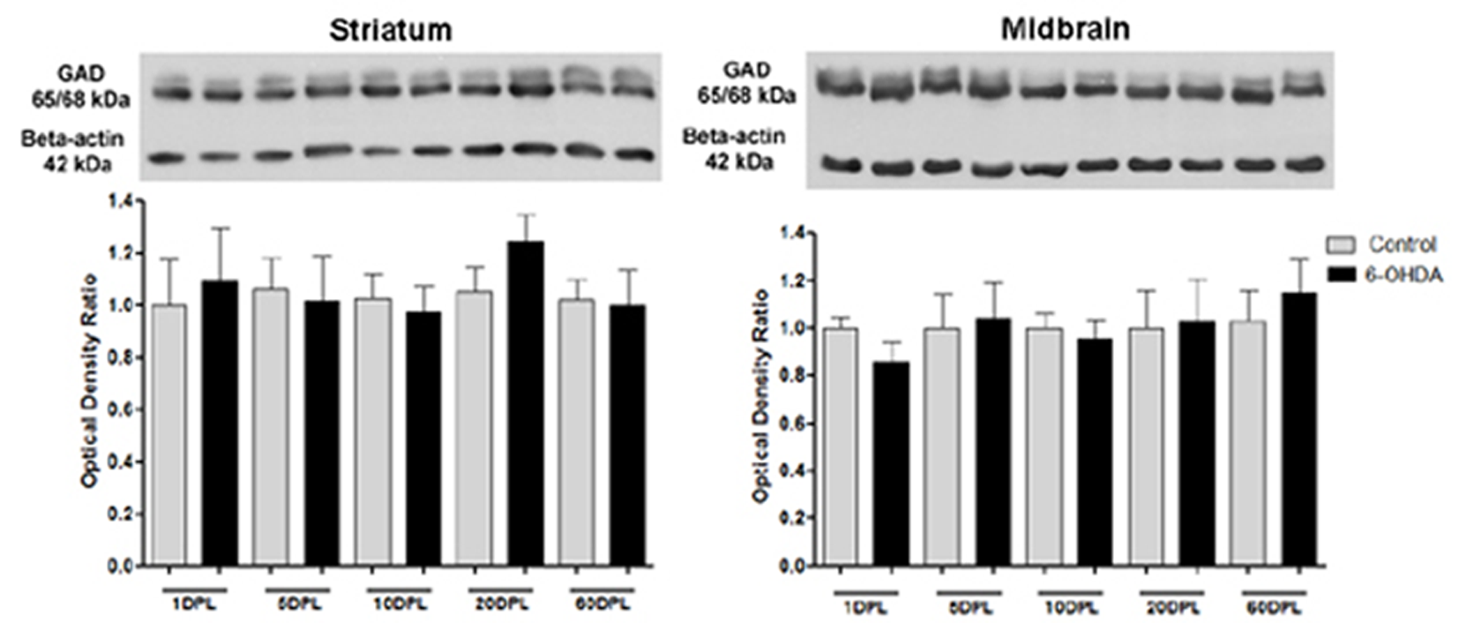

Supplement: Figure S1 — Temporal analysis of GAD levels in basal ganglia of PD-induced rats. Semi-quantitative analysis from immunoblotting. Mean ratio of GAD densitometry density data in relation to beta-actin, comparing the experimental with control side from each DPL. Statistic analysis by Paired Student t test. (TIF) [file pone.0076874.s001.tif]

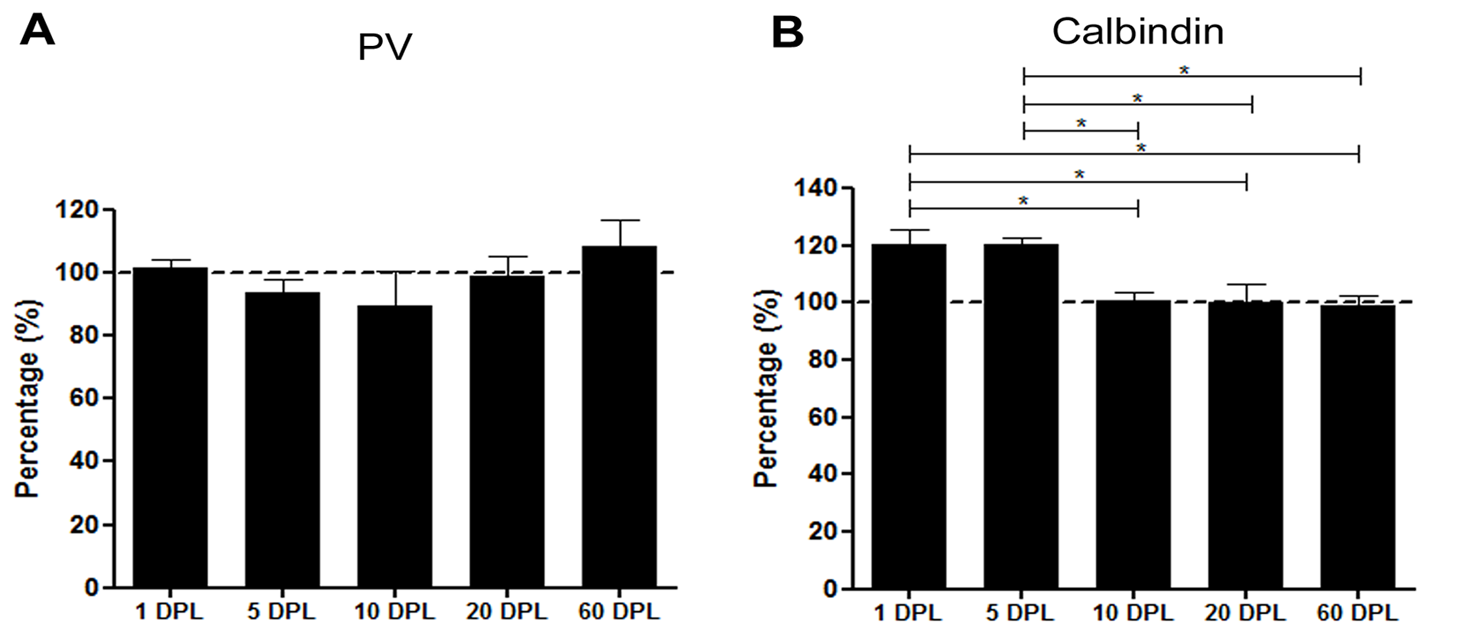

Supplement: Figure S2 — Semi-quantitative analysis of calbindin and parvalbumin staining in basal ganglia of PD-induced rats. The quantification is represented by percentage in the striatum of rats submitted to unilateral intrastriatal injections of 6-OHDA after 1, 5, 10, 20 and 60 days post-lesion (DPL). (A) Parvalbumin (PV) and (B) Calbidin. Comparison between the percentage of experimental sides calculated from the control side to each group (dashed line). Statistical analysis by ANOVA (one way) with Tukey post-test. *p <0.05, **p<0.01 and *** p <0.001. (TIF) [file pone.0076874.s002.tif]
